# Why might members of racially minoritized groups seek anonymity when interacting with White people online? Codeswitching, emotional labour and burnout

**DOI:** 10.1111/bjso.70060

**Published:** 2026-03-08

**Authors:** Lewis Nitschinsk, Melinda Hewett, Audree Grand'Pierre, Michael Thai, Fiona Kate Barlow

**Affiliations:** ^1^ School of Psychology University of Queensland Brisbane Queensland Australia

**Keywords:** anonymity, burnout, codeswitching, emotional labour, intergroup contact, interracial contact, online

## Abstract

People can alter the nature of online intergroup interactions by becoming anonymous. Across three studies (*N* = 1107), we surveyed Black (Studies 1–3) and White (Study 2) participants in majority‐White nations. We argue that Black people living in these countries face substantial pressures in interracial interactions, and that responses associated with the performative pressures of contact might predict a desire for anonymity in interracial settings online. We operationalized these responses in three distinct yet related ways: codeswitching (adjusting language or behaviour), emotional labour (suppressing negative and displaying positive emotions) and experiencing burnout from intergroup contact. As proposed: (1) Black participants who engaged in more codeswitching and emotional labour, and who felt more burned out when interacting with White people, were more likely to seek anonymity in an interracial interaction; (2) Black participants were more likely than White participants to engage in codeswitching and emotional labour, to feel burned out from interracial contact, and, in turn, to seek anonymity in interracial interactions; and (3) stigma consciousness and perceived discrimination partly explained the relationship between codeswitching, emotional labour, and burnout and seeking anonymity. Our findings elucidate how group processes might affect whether members of racially minoritised groups might seek anonymity online.

## INTRODUCTION

When racial minority and majority group members interact, both parties are aware of the risks of being negatively stereotyped or rejected because of their group membership (Bergsieker et al., 2010; Shelton, 2003; Shelton & Richeson, 2005). Even if an interaction ultimately proves positive, it often unfolds against a backdrop of intergroup anxiety and tension. More broadly, some people try to avoid interracial contact altogether, living segregated lives in otherwise integrated spaces (Barlow et al., 2009; Dixon et al., 2020, 2022, 2023; Paolini et al., 2024). Our research extends this literature by examining how these dynamics may operate in online contexts, where avoidance‐adjacent affordances such as anonymity are readily available. We advance the argument that online anonymity may be particularly appealing to some racially minoritized group members living in majority‐White societies. Specifically, we suggest that, against the backdrop of racism and marginalization, interracial interactions often entail heightened performative pressures, and that anonymity may be sought as a means of alleviating or managing these demands. Across three studies, we examine whether three constructs that index such performative pressures during interracial contact—codeswitching, emotional labour and interaction‐related burnout—are associated with a greater desire for anonymity when Black participants anticipate interacting with a White stranger online.

### Online anonymity and interracial contact

Most intergroup contact research has focused on face‐to‐face interactions (Pettigrew & Tropp, 2006), which are often casual, brief and embedded in public spaces such as cafés or public transport (Dixon et al., 2005; Schäfer et al., 2021). However, interracial contact also increasingly occurs in online environments (Imperato et al., 2021). Compared with face‐to‐face contexts, online settings reduce practical barriers to engagement and give people more control over how and when they engage with others (Amichai‐Hamburger & McKenna, 2006). These features may allow people greater flexibility in how they navigate online interracial interactions compared to face‐to‐face interactions (Pereira da Costa et al., 2024; Tropp et al., 2022).

One way this increased flexibility occurs is through the affordances that online environments provide. One such affordance is anonymity. Anonymity has long been understood to influence human behaviour. Early theorists argued that anonymity can lead to feelings of deindividuation, whereby people enter a state of reduced self‐awareness and ‘lose’ their self‐identity (Festinger et al., 1952; Zimbardo, 1969). More recent theories, most notably the social identity model of deindividuation effects (SIDE), refine early deindividuation accounts by arguing that anonymity does not reduce social influence but instead changes its basis. When individuals are anonymous, individuating cues that support personal identity and idiosyncratic self‐standards become less salient, weakening the role of personal norms and reputation concerns in guiding behaviour (Postmes & Spears, 1998; Reicher et al., 1995; Spears & Lea, 1994). In their place, social category memberships become more prominent reference points for self‐definition and for construing others, such that people are more likely to perceive both themselves and interaction partners in terms of shared social category memberships.

SIDE further proposes that anonymity reshapes group processes by altering visibility, accountability and power within an interaction (Klein et al., 2007; Reicher et al., 1995). Reduced personal visibility weakens the link between behaviour and individual evaluation, while diffuse accountability shifts anticipated consequences away from the individual. Anonymity may also recalibrate power relations by limiting the capacity for dominant group members to monitor or sanction identifiable individuals. Together, these changes can amplify group‐normative influence when social identities are salient or alternatively attenuate the interpersonal costs associated with deviating from expected behavioural standards.

SIDE has been commonly used to explain the results of anonymity and deindividuation research, much of which occurred during the early years of computer‐mediated communication, when online interactions were largely anonymous by default (Postmes & Spears, 1998). Contemporary online environments, however, often allow individuals to choose whether they wish to be anonymous or identifiable. For example, social media platforms such as *Instagram* and multiplayer online games such as *Roblox* allow users to modify names or profile images, toggle voice settings or maintain multiple profiles. Anonymity therefore functions not merely as a situational feature of online interaction, but as a strategic tool that individuals can use to shape how they want to engage (or not engage) with others. Consistent with the idea that people can use anonymity strategically, past research has found that people are motivated to seek anonymity for a variety of reasons, including to behave more selfishly, more altruistically, to self‐express or to engage in toxic behaviours (Nitschinsk, Tobin, & Vanman, 2025; Nitschinsk, Tobin, Varley, & Vanman 2025).

Anonymity may also allow people to obscure or downplay cues that signal racial identity, thereby reducing the intergroup character of an interaction (effectively shutting off intergroup performative pressures). Alternatively, anonymity may render people personally non‐identifiable, potentially attenuating concerns about downstream social or reputational consequences if they choose not to engage in codeswitching or emotional regulation. In either case anonymity should be appealing insofar as it may offer relief from the performative pressures that often accompany interracial interactions. This relief may be particularly important for racially minoritized group members in predominantly White societies, who face heightened risks of prejudice and discrimination during interracial interactions.

In the following section, we review research on why interracial interactions can be psychologically burdensome for racially minoritized group members. We note, however, that our aim is not to directly test the psychological mechanisms through which anonymity might reduce these pressures, but rather to examine whether individual differences in the experience of performative demands during interracial interactions predict preferences for anonymity in an online interracial context. We return to questions about what individuals believe anonymity affords, and what it may in fact achieve, in the General Discussion.

### The effort of interracial contact: Codeswitching, emotional labour and burnout

Cultural, social and systemic differences between racial groups can make contact difficult to navigate (Carey et al., 2022; Shelton et al., 2023; Taylor et al., 2021). For example, people often perceive outgroup members as less responsive to their values and perspectives than ingroup members (Livingstone et al., 2020). Here, we focus our attention on the experiences of Black people living in the United States, the United Kingdom, Canada, Australia and New Zealand. Although the historical and present‐day relationships between Black and White people from these nations are unique, all share commonalities including histories of colonization, slavery, displacement and continual systemic discrimination.

In majority‐White cultures, racial minority group members and Black people in particular, face the prospect of prejudice, racism and stereotyping during interracial interactions (Shelton et al., 2005; Spencer et al., 2016). Most interracial interactions are positive (Graf et al., 2014), but negative encounters may reinforce and entrench White prejudices (Barlow, Paolini, et al., 2012; Barlow, Sibley, & Hornsey, 2012; Hayward et al., 2017; Pinel, 1999; Williams et al., 1997) and contribute to racism, which can manifest in forms ranging from subtle discrimination to overt acts of violence (Sue et al., 2007). Even if racism is not explicit, Black Americans often worry that White Americans are concealing prejudice in interpersonal interactions (Kunstman et al., 2016). Perhaps relatedly, even well‐meaning White people can display White fragility, responding defensively or avoidantly to discussions of race or identity (DiAngelo, 2018; Ford et al., 2022). It is possible then that the combination of concerns around racism, stereotyping and White fragility may result in members of racially minoritized groups feeling pressure to ensure that their interactions with White people remain positive (for a similar argument see Bergsieker et al., 2010).

Communication accommodation theory argues that people strategically adjust their verbal and nonverbal communication to reduce or magnify communicative differences between themselves and others (Giles, 2008). This theory situates such adjustments within asymmetric power relations, whereby members of lower‐status groups are more likely to accommodate to higher‐status interlocutors to manage impressions, secure social acceptance or avoid negative evaluation. In interracial interactions, these accommodative behaviours may take the form of codeswitching, whereby individuals modify their language use, speech patterns or behavioural presentation to optimize the comfort of, or increase acceptance from, their interaction partners (Anicich & Hirsh, 2017; McCluney et al., 2021; Molinsky, 2007).

Codeswitching has long been documented as a strategy used by members of racial minority groups to navigate majority‐White social and professional contexts (e.g. Black Americans using White‐sounding names on résumés or reducing the use of colloquialisms; Kang et al., 2016; Koch et al., 2001; Scott, 2013). Consistent with communication accommodation theory's emphasis on audience design and impression management, racially minoritized individuals may also regulate emotional expression during interracial interactions, suppressing negative affect and performing positivity as a form of emotional labour (Evans & Moore, 2015; Grandey et al., 2019). Such emotional labour may reflect efforts to pre‐empt stereotyping or sanction, for example avoiding being perceived as angry, a stereotype disproportionately applied to Black men (Evans & Moore, 2015).

Due to the stress and pressures of interracial contact, both racial minority and majority group members likely engage in some level of codeswitching and emotional labour during interracial interactions. However, we suggest that minority group members are more likely to engage in these behaviours more often than majority group members. This is because, in an unequal society, racial minority group members face greater psychological and social costs when interracial interactions go poorly.

Stepping back, facing prejudice or feeling pressure to behave inauthentically may not only lead to performative behaviours, but it can also be exhausting. As a result, we suggest that separate from engaging in codeswitching or emotional labour, some people from racially minoritized groups may feel generally burned out from interacting with White people. Burnout is often studied in organizational contexts, where prolonged exposure to stressful work environments can increase frustration and lead to various negative physiological and emotional outcomes (Brotheridge & Grandey, 2002; Martínez‐Iñigo et al., 2007; Pisaniello et al., 2012). Consistent with the idea that the interracial context might be associated with burnout for racial minority group members, past work has found that Black mental health professionals who report having experienced more racial discrimination also tend to report more personal, work related and overall burnout (Brown & Homan, 2024). Qualitative work also shows that racial justice advocates of colour who have experienced burnout often attribute it to the racism of White activists (Gorski & Erakat, 2019).

### The current research

In sum, drawing on the literature reviewed above, we propose that members of racially minoritized groups are more likely than White people to engage in performative behaviours and experience burnout during interracial contact. As a result, racial minority group members may be more likely to seek anonymity in online interracial interactions, potentially as a means of reducing or avoiding these pressures. We test these predictions across three studies, operationalizing responses associated with the performative pressures of contact in three distinct yet interrelated forms: codeswitching, emotional labour and burnout from interactions with White people. Each is tested as an individual predictor of the desire for anonymity in interracial online interactions, with our analyses examining patterns across studies to assess which (if any) emerge as the strongest correlates.

In all studies we recruit participants living in majority‐White nations, including the United States, the United Kingdom, Canada, Australia and New Zealand. In Study 1, we focused solely on Black participants, who on average face substantial prejudice and discrimination and performative pressures in majority‐White nations. In Study 2, we tested whether Black participants reported higher levels of codeswitching, emotional labour and burnout than did White participants, and whether any differences explained differences in anonymity seeking. In Study 3, we returned our focus to Black participants and investigated whether stigma consciousness and perceived everyday discrimination were associated with codeswitching, emotional labour and burnout, and through this, seeking anonymity in an interracial interaction. All studies received ethics approval and were pre‐registered. For each study we pre‐registered the studies design, planned sample size, inclusion/exclusion criteria and planned analyses. Deviations from the pre‐registrations and exploratory analyses are noted within the article. Links to all pre‐registrations are available in the supplemental material document. Sensitivity analyses were conducted using G*Power (Faul et al., 2007). Data analyses were conducted using either R or SPSS (R Core Team, 2022).

## STUDY 1

Using a vignette design, Study 1 investigated whether codeswitching, emotional labour and burnout were associated with Black participants choosing to seek anonymity in an online encounter with either a Black person or a White person. We had several pre‐registered predictions. First, we predicted that Black participants would be more likely to seek anonymity when interacting with a White person compared to interacting with a Black person. Second, we predicted that participants who reported engaging in more codeswitching or emotional labour, or those who felt more burned out when interacting with White people in general, would be more likely to seek anonymity in an interaction with a White person. We also controlled for social anxiety, as past research has shown that people who are socially anxious are more likely to seek anonymity to interact with others (Nitschinsk, Tobin, Varley, & Vanman, 2025).

### Method

#### Participants and design

A sensitivity analysis for a linear multiple regression (fixed model, single regression coefficient: alpha = .05, predictors = 4) revealed that our sample of 314 participants would provide 95% power to detect small to medium effect sizes (*f* ^2^ = .04). We recruited 349 participants. Thirty‐five participants were excluded for failing to pass either an attention check or a manipulation check (final *N* = 314). We recruited Prolific workers in the United States, the United Kingdom, Canada, Australia and New Zealand. We screened participants so that only participants who said they were Black in response to the question ‘*What ethnic group do you belong to?*’ could complete the study. This pre‐screen, asked of all Prolific users, includes six response options: White, Black, Asian, Mixed, Other and Prefer not to say. For demographic statistics see Table 1. To prevent repeated participation across studies, Prolific's exclusion settings were used so that participants in Study 1 could not take part in Studies 2 or 3 (Study 2 participants were also excluded from Study 3).

**TABLE 1:** Demographic statistics for Studies 1, 2 and 3.

		Study 1	Study 2	Study 3
Number of participants		314	560	235
Gender	Men	163	323	141
	Women	148	227	94
	Nonbinary	2	9	0
	Prefer not to say	1	1	0
Age	Mean	37.9	38.5	37.03
	Standard deviation	11.66	12.2	11.6
	Range	19–77	18–86	18–76
Education	High school or less	20	76	28
	University education	200	343	149
	Post‐graduate education	94	141	58
Relationship status	Single	120	205	100
	Dating	75	97	46
	Married	91	214	69
	Divorced	16	30	15
	Widowed	3	9	2
	Prefer not to say	9	5	3

#### Measures and procedures

Participants first completed scales assessing codeswitching and emotional labour towards White people. They were instructed: ‘*Reflecting on how you feel when you interact with White people, in general, please tick the box that best describes your experience*’. To assess codeswitching, we developed eight items based on qualitative responses in past research (e.g. ‘I try to smile more’, ‘I alter the way I speak’; Evans & Moore, 2015; McCluney et al., 2021).^1^ Emotional labour was measured with the three‐item surface acting subscale of the Emotional Labour Scale (e.g. ‘I resist expressing my true feelings’, ‘I pretend to have emotions that I don't really have’; Brotheridge & Lee, 2003). Both were rated on 7‐point scales (1 = *Strongly Disagree*, 7 = *Strongly Agree*).

To assess burnout, participants first read the following sentence: *Reflecting on how you feel after spending time with White people, please tick the box that best describes your experience*. Then participants completed an adapted version of the eight‐item Maslach burnout inventory to reflect contact with White people (e.g. ‘I feel frustrated after spending time with White people’, ‘Interacting directly with White people puts too much stress on me’; Maslach et al., 1997). Items were answered on a 5‐point scale (1 = *Never*, 5 = *Always*). To assess social anxiety, we used the six‐item social anxiety subscale from the revised self‐consciousness scale (0 = *not like me at all*, 3 = *a lot like me*; Scheier & Carver, 1985). Participants were then asked to read the following scenario:

…you are a student attending your first year of university. During the year, you decide to sign up to participate in a study for course credit. When you arrive, the researcher takes you to an individual computer lab where you will complete the study. The researcher tells you that this study will be about how people form impressions of one another.

Within the scenario, participants were told they were about to have a conversation with another person via an online video chat. Participants were randomly assigned to interact with either a White person or a Black person. Participants were then given the choice whether they would participate anonymously or identifiably in the scenario. Identifiability meant their camera would be turned on and their name would be visible whereas anonymity meant their camera would be turned off and their name would not be visible. Participants were told that the person they were about to have a conversation with would be identifiable.^2^

Next, participants completed a manipulation check confirming they encoded their partner's race, followed by two attention checks: (1) selecting ‘Strongly Agree’ to a target item and (2) typing the phrase ‘I am paying attention’. As pre‐registered, those who failed either check or the manipulation check were excluded. Finally, participants reported demographic information and completed additional questionnaires not relevant to this paper (see Supplemental Materials).

#### Transparency and openness

For all studies, data, materials and analysis code have been made publicly available using the Open Science Framework. The link can be accessed here: https://osf.io/fx95b/overview?view_only=fe54b7630e634652b57ed0107c1e71e6.

#### Data analysis

We first conducted an independent‐samples t‐test to examine whether seeking anonymity differed by interaction partner race. We then performed bivariate correlations between all continuous variables. Point biserial correlations were conducted between predictor variables and choosing to be anonymous in same‐race or cross‐race interactions. Next, we ran three logistic regressions. Each regression included social anxiety, partner race (−1 = same race, 1 = different race), one focal predictor (either codeswitching, emotional labour or burnout) and the interaction term between the focal predictor and partner race. Predictor variables were entered simultaneously. Continuous predictors were mean‐centred. The dependent variable was whether participants chose anonymity or identifiability. For significant interactions, we probed simple slopes at ±1SD for each predictor and at each level of partner race, applying Tukey adjustments to control for Type I error.

#### Model selection

In all studies we pre‐registered regressions to examine emotional labour, codeswitching and burnout as individual predictors of seeking anonymity. We did this as we wanted to look at how different responses to the performative pressures of interracial contact might be associated with seeking anonymity, operationalizing these responses in three ways: codeswitching, emotional labour and burnout. We anticipated each of these phenomena to be highly correlated and thus felt it was most appropriate to look at associations in separate models, looking for consistencies in patterns over our three studies. For completeness, however, in each study and for the mega‐analysis we also ran additional regressions and mediations where emotional labour, codeswitching and burnout, as well as their interactions with partner race (when applicable) were included simultaneously. These analyses are available in the supplemental materials.

### Results

For descriptive statistics, scale reliabilities see Table 2. We found participants were not more likely to see anonymity in the cross‐race condition (42.9%) compared to the same‐race condition (39.1%, *t* = −.069, *p* = .487). In cross‐race interactions, burnout, codeswitching and social anxiety were positively associated with seeking anonymity (see Table 2). In same‐race interactions, no predictor variable was associated with seeking anonymity. Codeswitching, emotional labour and burnout were all positively associated with each other (*r*'s = .26–.54, *p*'s < .001).

**TABLE 2:** Descriptive statistics, scale reliabilities and point biserial correlations between focal predictor variables and seeking anonymity in cross‐race and same‐race interactions.

	*M (SD)*	α	Anonymity cross‐race	Anonymity same‐race
1. Burnout	1.86 (0.97)	.96	.20**	−.11
2. Codeswitching	4.65 (1.31)	.87	.23**	−.03
3. Emotional Labour	3.36 (1.68)	.85	.13	.02
4. Social Anxiety	1.40 (0.86)	.88	.16*	.15

*Note*: Cross‐race *N =* 163. Same‐race *N* = 151. Choosing to be anonymous or identifiable is coded as 1 = Anonymous, 0 = Identifiable. *p < .05, **p < .01, ***p < .001.

In our regression models, seeking anonymity did not differ based on the interaction partner (same‐race or cross‐race; see Table 3). Social anxiety was positively associated with choosing to be anonymous in the vignette, whereas codeswitching, emotional labour and burnout were not.

**TABLE 3:** Logistic regressions for partner race, social anxiety, codeswitching, emotional labour and burnout as predictors of choosing to be anonymous or identifiable during a social interaction.

Predictors	Wald	Odds ratio	95% CI
Panel A: Burnout			
Intercept	−.84	.70**	.55–.88
Partner Race	.09	1.09	.87–1.37
Burnout	.04	1.04	.81–1.34
Social Anxiety	.35	1.42*	1.08–1.88
Partner Race * Burnout	.33	1.40**	1.10–1.80
Panel B: Codeswitching			
Intercept	.90	.40***	.25–.64
Partner Race	.08	1.08	.86–1.36
Codeswitching	.15	1.16	.96–1.41
Social Anxiety	.38	1.46**	1.11–1.93
Partner Race * Codeswitching	.25	1.28**	1.07–1.56
Panel C: Emotional Labour			
Intercept	−.85	.69**	.55–.87
Partner Race	.08	1.09	.87–1.37
Emotional Labour	.04	1.04	.89–1.20
Social Anxiety	.35	1.41*	1.07–1.88
Partner Race * Emotional Labour	.07	1.07	.94–1.27

*Note*: Partner Race is coded as −1 = Black interaction partner, 1 = White interaction partner. Choosing to be anonymous or identifiable is coded as 1 = Anonymous, 0 = Identifiable. *p < .05. **p < .01. ***p < .001.

Abbreviation: CI, confidence interval.

**FIGURE 1:**
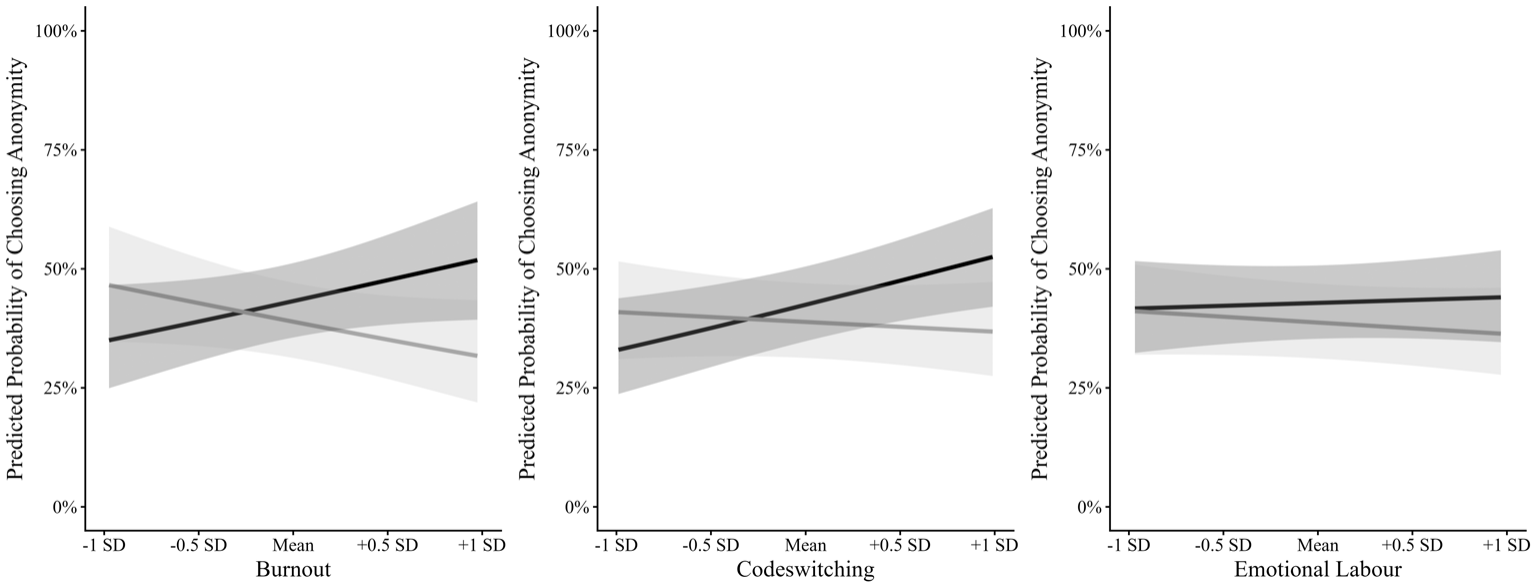
The relationship between interacting with a White or a Black person and burnout, codeswitching, and emotional labour on choosing to be anonymous (Study 1). Grey = Same‐race interaction, Black = Cross‐race interaction. The y‐axis is a dichotomous dependent variable. Figures for burnout and codeswitching display a significant interaction. Shaded areas indicate 95% confidence intervals.

Beyond these main effects, we observed a significant interaction between burnout and partner race (see Panel A of Table 3). Probing this interaction by treating partner race as the moderator indicated that the association between burnout and seeking anonymity differed in direction across interaction contexts; however, neither simple slope reached statistical significance (same‐race: *b* = −.58, *p* = .178; cross‐race: *b* = .75, *p* = .083; see Figure 1). Decomposing the interaction the other way (i.e. looking at burnout as the moderator) participants who reported feeling more burned out after interacting with White people (+1SD) were more likely to seek anonymity in cross‐race interactions compared to same‐race interactions (*b* = .84, *p* = .031). For participants who reported feeling less burned out while interacting with White people (−1SD) the difference was not significant (*b* = −.50, *p* = .283).

We also found a significant interaction between codeswitching and partner race (see Panel B of Table 3 and Figure 1). When looking at interaction partner race as the moderator, we found that participants reported engaging in more codeswitching while interacting with White people and were more likely to seek anonymity in cross‐race interactions (*b* = .79, *p* = .014), but not same‐race interactions (*b* = −.20, *p* = .659). When looking at codeswitching as the moderator, we found that participants who reported engaging in more codeswitching while interacting with White people (+1SD) were not more likely to seek anonymity in cross‐race interactions compared to same‐race interactions (*b* = .65, *p* = .056). For participants who reported engaging in less codeswitching while interacting with White people (‐1SD), the difference was also not significant (*b* = −.35, *p* = .463), but the estimates were in different directions. There was no interaction between partner race and emotional labour.

### Discussion

Although Black participants were not generally more likely to seek anonymity in cross‐race interactions compared to same‐race interactions, consistent with our second prediction we found that the association between burnout and anonymity, and between codeswitching and anonymity, depended on the race of the interaction partner. For burnout, the slopes relating burnout to anonymity were estimated in opposing directions in same‐race and cross‐race interactions, though neither slope reached statistical significance. In contrast, greater engagement in codeswitching was associated with a higher likelihood of seeking anonymity when interacting with a White but not a Black interaction partner online. Additionally, participants who reported higher burnout or engaged in more codeswitching were more likely to seek anonymity in cross‐race than same‐race interactions, whereas those lower in burnout or engaged in less codeswitching showed no difference.

## STUDY 2

In Study 2 we investigated whether seeking anonymity in same‐race or cross‐race interactions differed for Black and White people living in majority‐White nations. The frequency and potential consequences of interracial contact differ for Black and White people. Black people living in majority‐White nations, for example, face interpersonal and systemic prejudice and discrimination in a way that White people simply do not. Consequently, we predict that Black participants, compared to White participants, will be more likely to engage in codeswitching and emotional labour during interracial contact, experience more burnout from interracial contact and be more likely to seek anonymity in an anticipated interracial interaction.

Having said this, interracial interactions can still elicit anxiety for White people, particularly around appearing prejudiced (Apfelbaum et al., 2008; Kunstman et al., 2016). This anxiety may lead White people to modify their behaviour to appear more likeable, which can in turn be exhausting (Bergsieker et al., 2010; Butz & Plant, 2009). As such, while we expect mean differences in codeswitching, emotional labour and burnout to the extent that both Black and White participants report experiencing each variable (in particular codeswitching and burnout, which were significant predictors in Study 1), we predict they will be more likely to seek anonymity in the scenario.

We therefore first test a model whereby group membership predicts increased burnout, and through this, an increased desire to be anonymous; that is, we propose that Black participants will report engaging in more burnout when interacting with White people than vice versa, and that this will explain a general tendency for Black people to prefer anonymity when engaging in cross‐race online contact, compared to same‐race online contact. We then test the same model twice more, considering codeswitching and emotional labour.

Next, we follow these analyses up with a series of tests of moderation. As noted above, the racialized pressures in interracial interactions are more severe and more consequential for Black relative to White people. It is therefore possible that codeswitching, emotional labour and burnout predict an increased desire for anonymity in cross‐race but not same‐race interactions more consistently for Black relative to White people. We explore this prediction by testing each two‐way interaction (between race of interaction partner and either codeswitching, emotional labour, or burnout) separately for Black and White participants.

### Method

#### Participants

A sensitivity analysis for a linear multiple regression (fixed model, single regression coefficient: alpha = .05, predictors = 4 revealed that our sample of 560 participants would provide 95% power to detect small to medium effect sizes (*f* ^2^ = .02)). We initially recruited 599 participants. Thirty‐nine participants were excluded for failing to pass either an attention check or a manipulation check (final *N* = 560). We recruited Prolific workers in the United States, the United Kingdom, Canada, Australia and New Zealand. We screened participants so that only participants who said they were Black or White in response to the question ‘*What ethnic group do you belong to?*’ could complete the study. For demographic statistics see Table 1.

#### Measures and procedures

The measures and procedures were identical to Study 1. For Black participants, all items relating to codeswitching, emotional labour and burnout were related to engaging with White people and vice versa.

#### Data analysis

First, we compared mean differences in seeking anonymity, burnout, codeswitching and emotional labour between White and Black participants. We then performed bivariate correlations between all continuous variables separately for White and Black participants. Point biserial correlations were also conducted between predictor variables and choosing to be anonymous in same‐race or cross‐race interactions.

Second, we conducted three moderated mediations using Model 15 of the PROCESS macro (Hayes, 2012), race (−1 = Black, 1 = White) was the predictor, either codeswitching, emotional labour or burnout served as the mediator in its own model, partner ethnicity (−1 = same race, 1 = interracial) moderated the *b* and *c* paths, social anxiety was a covariate and seeking anonymity the dependent variable. Note that these moderated mediations reflect the most direct test of our theoretical arguments but due to error were not pre‐registered.

Third, we ran logistic regressions identical to those in Study 1, separately for White and Black participants. In our pre‐registration we planned on testing the three‐way interaction between participant race, partner race and either burnout, codeswitching, or emotional labour, but ultimately did not include these analyses in the main text due to limited statistical power. Our total sample of 560 participants is too small to robustly test a three‐way interaction that may follow an attenuation or knockout pattern. Such a pattern would suggest that a predictor (e.g. codeswitching) affects anonymity only for one group (e.g. Black participants interacting with a White partner), and not at all for others, essentially ‘knocking out’ the effect in other subgroups. Detecting such nuanced patterns requires very large sample sizes (potentially up to 14 times larger than ours) to achieve adequate power (Baranger et al., 2023; da Silva Frost & Ledgerwood, 2020).

### Results

For descriptive statistics and correlations, see Table 4. Black participants engaged in more codeswitching and emotional labour, experienced more burnout and sought anonymity more often than did White participants in cross‐race interactions. When examining the correlations split by participant race, burnout and emotional labour were positively associated with seeking anonymity in cross‐race interactions for both White and Black participants. Additionally, codeswitching and social anxiety were positively associated with seeking anonymity in cross‐race interactions for Black participants only.

**TABLE 4:** Means and standard deviations of focal variables for Black and White participants and point biserial correlations between focal predictor variables and seeking anonymity in cross‐race and same‐race interactions for White and Black participants.

	White participants			Black participants		
	Mean (SD)	Cross‐race	Same‐race	Mean (SD)	Cross‐race	Same‐race
Same‐Race	.45 (.50)^a^			.45 (.50)^a^		
Cross‐Race	.54 (.50)^a^			.32 (.57)^b^		
Burnout	1.95 (.91)^a^	.21*	.15	1.28 (.61)^b^	.18*	−.14
Codeswitching	4.51 (1.30)^a^	.12	.06	3.72 (1.15)^b^	.17*	−.03
Emotional Labour	3.25 (1.56)^a^	.24*	.06	2.37 (1.16)^b^	.28***	−.03
Social Anxiety	1.39 (.88)^a^	.09	.16	1.83 (.82)^b^	.19*	.19*

*Note*: Cross‐Race, seeking anonymity in a cross‐race interaction. Same‐Race, seeking anonymity in a same‐race interaction. Mean is shown outside of brackets. Standard deviation is shown inside brackets. Variations in superscripts within rows indicate that means are significantly different. For point biserial correlations, choosing to be anonymous or identifiable is coded as 1 = Anonymous, 0 = Identifiable. *p < .05. **p < .01. ***p < .001.

Abbreviation: SD, standard deviation.

#### Mediation analyses

**FIGURE 2:**
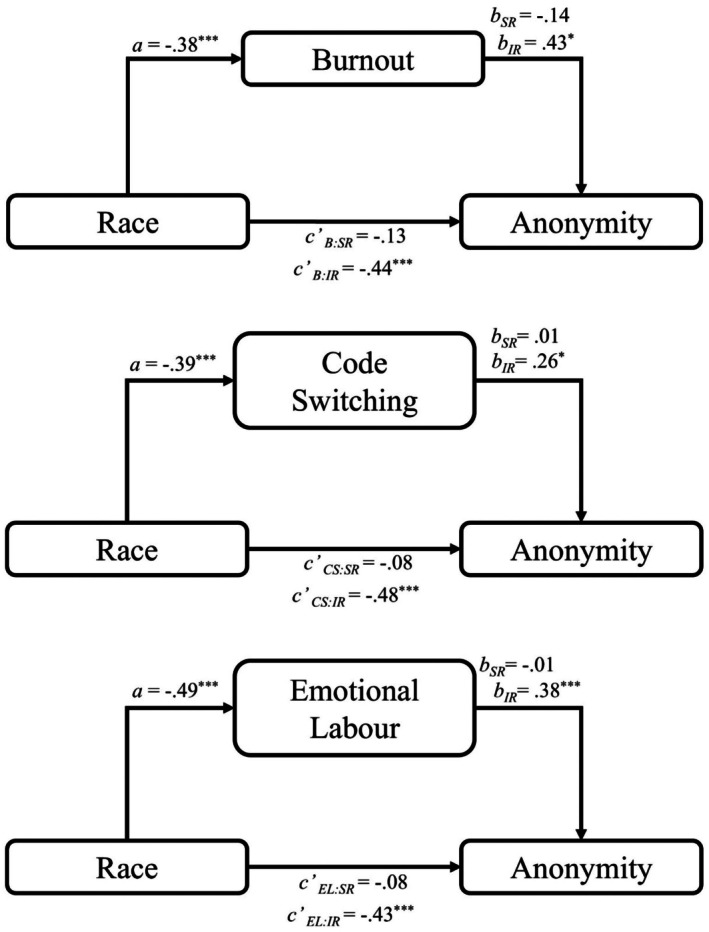
Moderated mediation models for the relationship between race and seeking anonymity when interacting with someone from the same racial group or a different racial group via either codeswitching, emotional labour or burnout. Race is coded as 1 = White, −1 = Black. Choosing to be anonymous or identifiable is coded as 1 = Anonymous, 0 = Identifiable. Social anxiety is included as a covariate for all pathways. B, burnout, CS, codeswitching, EL, emotional labour. SR, same race, IR, interracial. *p < .05. **p < .01. ***p < .001.

The moderated mediation analyses aimed to test a pattern consistent with the proposition that Black people seek more online anonymity in other race interactions than do White people in part because they experience more pressures during contact than do White people. This pattern is exactly what we found. Black participants reported higher burnout, codeswitching and emotional labour than White participants, and in turn, each variable was positively associated with seeking anonymity in cross‐race (but not same‐race) interactions. Indirect effects emerged only in cross‐race interactions; burnout, codeswitching and emotional labour each independently helped to explain the tendency for Black (relative to White) people to seek online anonymity in cross‐race interactions (see Figure 2 for full models and Table 5 for indirect effects).

**TABLE 5:** Conditional indirect effects for the relationship between race and seeking anonymity or identifiability when interacting with someone from the same racial group or a different racial group via burnout, codeswitching and emotional labour.

Mediator	Condition	Effect	SE	95% CI
Burnout	Same‐Race	.01	.04	−.07–.09
	Cross‐Race	.10*	.05	.02–.20
Codeswitching	Same‐Race	−.01	.05	−.09–.09
	Cross‐Race	.19*	.05	.09–.30
Emotional Labour	Same‐Race	−.06	.06	−.19–.07
	Cross‐Race	.16*	.07	.03–.31

* Significant indirect effect.

#### Moderated regression analyses

We next aimed to test the relationship between codeswitching, emotional labour and burnout and seeking anonymity in either cross‐race or same‐race interactions for White and Black participants separately. We found that for Black participants there was a significant interaction between burnout and partner race (*Wald* = .36, *Odds Ratio* = 1.44, *p* = .010) and emotional labour and partner race (*Wald* = .22, *Odds Ratio* = 1.25, *p* = .008), but not between codeswitching and partner race (*Wald* = .18, *Odds Ratio* = 1.19, *p* = .071). In same‐race interactions, feeling more burnout while interacting with White people was negatively associated with seeking anonymity (*b* = −.834, *p* = .045), whereas in cross‐race interactions there was no association (*b* = .50, *p* = .336). Decomposing the interaction with burnout as the moderator found that feeling more burnout while interacting with White people (+1SD) was more likely to seek anonymity in cross‐race interactions compared to same‐race interactions (*b* = 1.13, *p* = .004), but no difference was found for people who felt less burnout (‐1SD; *b* = −.20, *p* = .815).

We also found that in same‐race interactions, emotional labour was not associated with seeking anonymity (*b* = −.308, *p =* .622) but was positively associated with seeking anonymity in cross‐race interactions (*b* = 1.07, *p* = .012). When emotional labour was treated as the moderator we found that Black participants who reported engaging in more emotional labour while interacting with White people (+1SD) were more likely to seek anonymity in cross‐race interactions compared to same‐race interactions (*b* = 1.13, *p* = .004), but no difference was found for people who engaged in less emotional labour (−1SD; *b* = −.26, *p* = .720).

For White participants, interaction terms were not significant; burnout, codeswitching and emotional labour during interracial interactions did not differentially predict seeking anonymity in same or other race interactions (*Wald'*s = .06–.15, *Odds Ratios* = 1.06–1.16, *p'*s = .164–.675). Refer to supplemental materials for complete models and simple slopes analyses for significant interactions.

### Discussion

Study 2 demonstrated that Black (relative to White) participants reported engaging in more codeswitching and emotional labour and experiencing greater burnout when engaging with White people. Black participants were also more likely to seek anonymity in cross‐race interactions than White participants. As anticipated, the former helped to explain the latter pattern: participant race indirectly predicted seeking anonymity in cross‐race interactions through burnout, codeswitching and emotional labour (in separate models).

When drilling down into the relationship between either codeswitching, emotional labour, or burnout and seeking anonymity for White and Black participants, we found little evidence that they were differentially associated with anonymity in cross‐race versus same‐race interactions for White participants. Taken together, these findings are consistent with our argument that the racialized pressures in interracial interactions are more severe and consequential for Black people, and that such performance concerns may be particularly meaningful for understanding why Black people living in majority‐White cultures seek online anonymity in interracial encounters.

Having established that anonymity seeking is most pronounced in cross‐race interactions with White partners, Study 3 shifts focus from identifying when this occurs to examining why some Black people are more likely than others to seek anonymity online. Up until this point, our argument has been that such pressures are the result of a prejudiced society. Accordingly, in our final study we focus exclusively on cross‐race interactions to test this possibility.

## STUDY 3

As stated earlier, Black people living in majority‐White countries face the prospect of prejudice and discrimination because of their race. These experiences are not uniform, however. Some individuals are more aware of the stigma attached to their racial identity (stigma consciousness; Pinel, 1999), while others experience greater levels of day‐to‐day discrimination (everyday discrimination; Williams et al., 1997). Put simply, we expect that people who are more conscious of stigma, or who face more day‐to‐day discrimination, will feel greater pressure to codeswitch and perform emotional labour in interracial interactions, and will generally feel more burned out when interacting with White people. In turn, these responses associated with the performative pressures of contact should predict seeking anonymity in online interracial interactions.^3^

### Participants and method

A sensitivity analysis for a linear multiple regression (fixed model, single regression coefficient: alpha = .05, predictors = 3) revealed that our sample of 235 participants would provide 95% power to detect small to medium effect sizes (*f* ^2^ = .07). Initially we recruited 252 participants. Seventeen participants were excluded for failing to pass either an attention check or a manipulation check (final *N* = 235). We recruited Prolific workers in the United States, the United Kingdom, Canada, Australia and New Zealand. We screened so that only participants who said they were Black in response to the question ‘*What ethnic group do you belong to?*’ could complete the study. For demographic statistics see Table 1.

The measures and procedures used in this study were identical to those in Studies 1 and 2. Additionally, we assessed stigma consciousness using the stigma consciousness scale (Pinel, 1999). Participants first read the following prompt: ‘*Please respond to the questions below regarding the extent to which stereotypes about Black people affect you and your interactions with White people*.’ They then completed 10 items assessing their perceived awareness of racial stigma (e.g. ‘Most people have a problem viewing Black people as equals’; 1 = Strongly Disagree, 7 = Strongly Agree).

To assess everyday discrimination, participants completed the everyday discrimination scale (Williams et al., 1997). The scale began with the question: ‘*In your day‐to‐day interactions with White people, how often do any of the following things happen to you?*’ Participants then indicated the frequency of nine different discriminatory experiences (e.g. ‘You are treated with less respect than other people are’; 1 = Never, 6 = Almost Every Day).

### Data analysis

Bivariate correlations were conducted as in Studies 1 and 2. Additionally, we pre‐registered a series of mediations. Stigma consciousness and everyday discrimination were included as predictor variables. Codeswitching, emotional labour or burnout were included as mediators in separate models. The dependent variable was whether participants chose to seek anonymity or identifiability in the scenario. Social anxiety was included as a covariate in all models.

### Results

Overall, 59% of participants chose to be anonymous in the scenario. Stigma consciousness was positively associated with burnout, emotional labour and seeking anonymity with a White person. Everyday discrimination was positively associated with codeswitching, emotional labour and burnout, but was not significantly related to seeking anonymity with a White person (see Table 6).

**TABLE 6:** Descriptive statistics, scale reliabilities and correlations between focal predictors in Study 3.

	M (SD)	α	1	2	3	4	5	6
1. Anonymity	.59 (.49)							
2. Burnout	1.86 (.97)	.96	.23***					
3. Codeswitching	4.65 (1.31)	.87	.01	.14*				
4. Emotional Labour	3.36 (1.68)	.85	.22***	.40***	.58***			
5. Social Anxiety	1.40 (.86)	.88	.21**	.31***	.16***	.35***		
6. Stigma Consciousness	4.76 (1.10)	.85	.16*	.52***	.08	.19**	.19**	
7. Everyday Discrimination	2.49 (1.05)	.93	.10	.55***	.23***	.27***	.18**	.50***

*Note*: *N* = 186, α = Cronbach's alpha. *M*, mean. *SD*, standard deviation. Point biserial correlations were conducted between predictor variables and choosing to be anonymous. Choosing to be anonymous or identifiable is coded as 1 = Anonymous, 0 = Identifiable. *p < .05. **p < .01. ***p < .001.

### Mediation analyses

**FIGURE 3:**
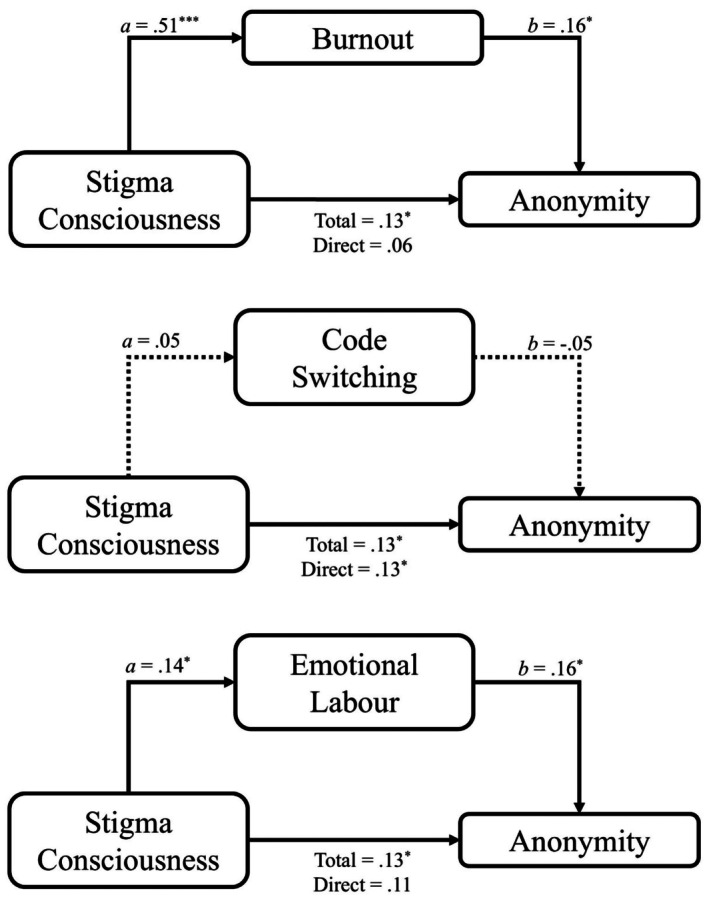
Mediation between everyday discrimination and codeswitching, emotional labour and burnout, on seeking anonymity when interacting with a White person. Social anxiety is included as a covariate for all pathways. Dotted lines indicate nonsignificant pathway. *p < .05. **p < .01. ***p < .001.

**FIGURE 4:**
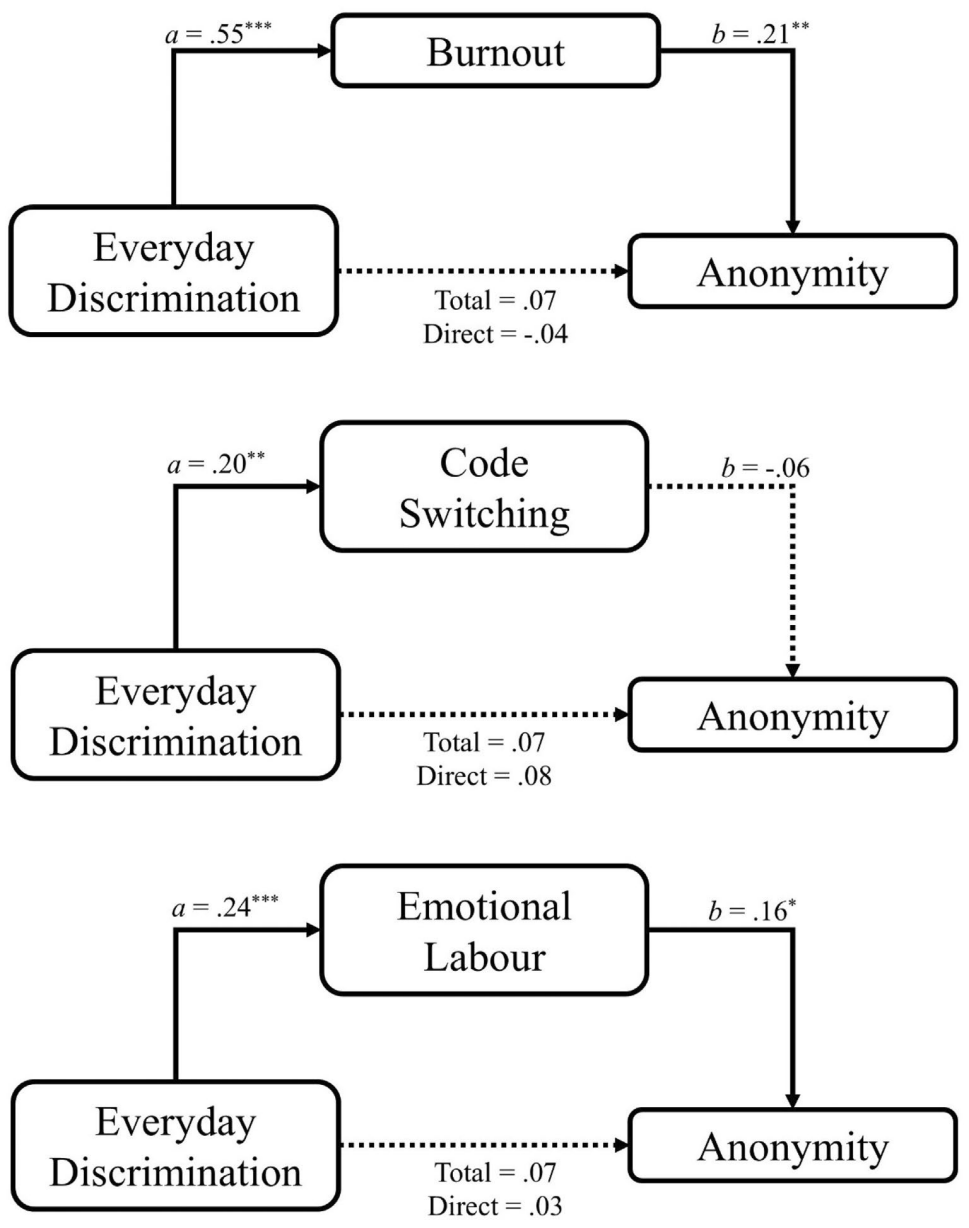
Mediation between everyday discrimination and codeswitching, emotional labour and burnout, on seeking anonymity when interacting with a White person. Social anxiety is included as a covariate for all pathways. Dotted lines indicate nonsignificant pathway. *p < .05. **p < .01. ***p < .001.

Burnout mediated the relationship between stigma consciousness and seeking anonymity when interacting with a White person (see Figure 3 and Table 7). Neither codeswitching nor emotional labour significantly mediated this relationship. Both burnout and emotional labour mediated the relationship between everyday discrimination and seeking anonymity, whereas codeswitching did not (see Figure 4).

**TABLE 7:** Indirect effects for the relationship between stigma consciousness and everyday discrimination and seeking anonymity or identifiability when interacting with a White person via burnout, codeswitching and emotional labour.

Mediator	Predictor	Effect	SE	95% CI
Burnout	Stigma consciousness	.08*	.04	.01–.15
	Everyday discrimination	.11*	.01	.03–.19
Codeswitching	Stigma consciousness	−.01	.01	−.01–.01
	Everyday discrimination	−.01	.06	−.04–.02
Emotional labour	Stigma consciousness	.02	.01	−.01–.05
	Everyday discrimination	.04*	.02	.01–.08

* Significant indirect effect.

### Discussion

Consistent with Study 1 and 2, we found that burnout was positively associated with seeking anonymity in a cross‐race interaction. Further, both stigma consciousness and everyday discrimination were positively associated with burnout, which, in turn, were associated with choosing anonymity in a cross‐race interaction. When assessing performative behaviours (emotional labour and codeswitching), results were more mixed. We found that emotional labour was positively associated with seeking anonymity in a cross‐race interaction, whereas codeswitching was not. Emotional labour also mediated the relationship between everyday discrimination and anonymity, though not between stigma consciousness and anonymity. Codeswitching did not mediate either relationship.

Interestingly, zero‐order correlations found that stigma consciousness and everyday discrimination were weakly related or un‐related to seeking anonymity in cross‐race interactions, respectively. While discrimination may be a distal contributor to seeking anonymity, more immediate concerns (e.g. the extent to which the interaction is likely to be exhausting) are more important to those deciding whether to be anonymous. Alternatively, there may be suppression at work; it is possible that perceived stigma and everyday discrimination are simultaneously associated with wanting to seek anonymity (through burnout) and wanting to confront difficult interracial interactions and seek identifiability, perhaps through increased ethnic identification (which is reliably associated with perceived discrimination; e.g. Branscombe et al., 1999; Barlow, Paolini, et al. 2012). Such possibilities could be examined in future work.

### Mega‐analysis

**FIGURE 5:**
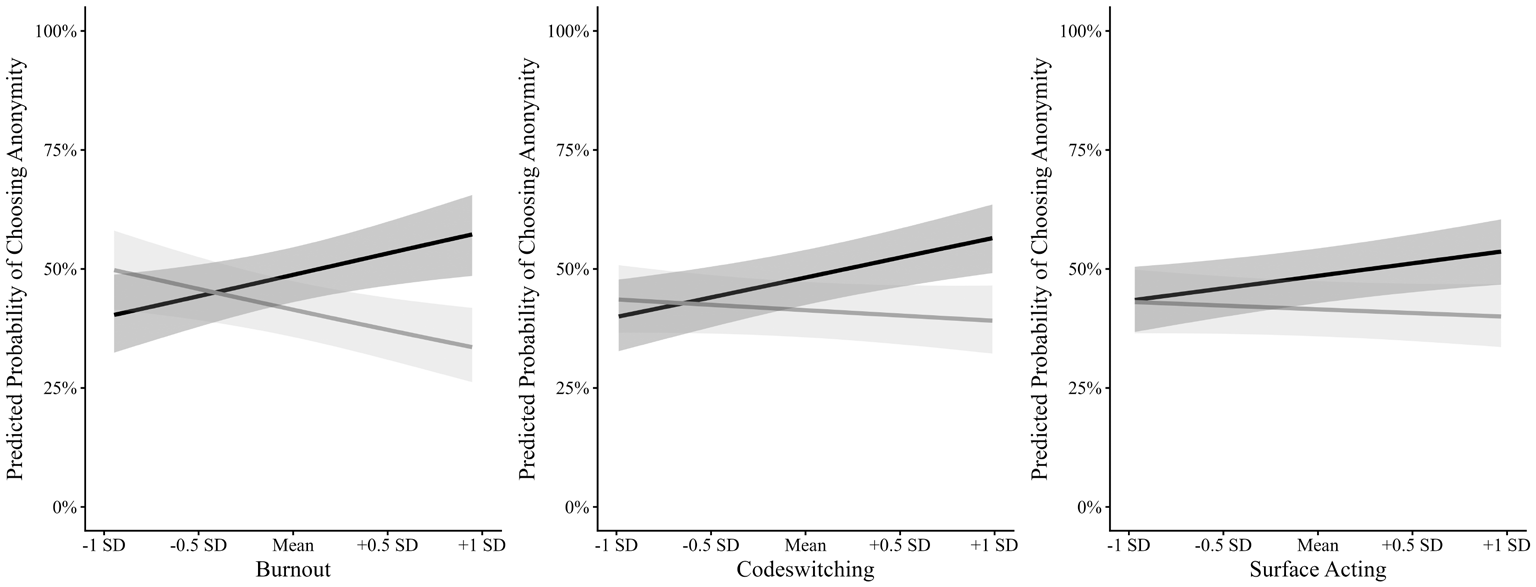
The relationship between interacting with a White or a Black person and burnout, codeswitching, and emotional labour on choosing to be anonymous for Black participants (Mega‐Analysis). Grey, same‐race interaction; Black, cross‐race interaction. The y‐axis is a dichotomous dependent variable. All figures denote a significant interaction. Shaded areas indicate 95% confidence intervals.

Elements of our design were repeated across studies. As such, we conducted a mega‐analysis pooling participant‐level data to better understand whether Black participants seek anonymity when interacting with White people compared to their own group, and whether this was moderated by either codeswitching, emotional labour or burnout (Burke et al., 2017; Curran & Hussong, 2009). We pooled participants from Study 1 and 2 (*N* = 587). We then ran mixed‐effects logistic regressions identical to Study 1, with study included as a random effect (see Table 8). We did not find a main effect of partner race, codeswitching, emotional labour or burnout on seeking anonymity. However, we found significant interactions between partner race and codeswitching, emotional labour and burnout, respectively. In all cases, we found that in cross‐race interactions burnout, codeswitching and emotional labour each predicted seeking anonymity in interactions with White people. In same‐race interactions, only burnout predicted a greater likelihood of seeking identifiability. See Table 9 for follow‐up tests. Breaking the interaction down the other way revealed that participants who reported more burnout, codeswitching and emotional labour when interacting with White people (+1 SD) were more likely to seek anonymity in cross‐race than in same‐race interactions, whereas participants who reported low levels of each variable (−1 SD) showed no difference (See Figure 5).

**TABLE 8:** Mega‐analytic mixed‐effects logistic regressions for partner race, social anxiety, codeswitching, emotional labour and burnout as predictors of Black participants choosing to be anonymous or identifiable during an interaction.

Predictors	Wald	Odds ratio	95% CI
Panel A: Burnout			
Intercept	−.19	.83	.64–1.06
Partner Race	.15	1.16	.98–1.37
Burnout	−.01	1.00	.82–1.21
Social Anxiety	.41	1.50***	1.23–1.85
Partner Race * Burnout	.35	1.42***	1.18–1.71
Panel B: Codeswitching			
Intercept	−.20	.82	.63–1.06
Partner Race	.14	1.15	.97–1.36
Codeswitching	.13	1.14	.99–1.30
Social Anxiety	.41	1.51***	1.24–1.84
Partner Race * Codeswitching	.21	1.24**	1.08–1.41
Panel C: Emotional Labour			
Intercept	−.19	.82	.63–1.07
Partner Race	.14	1.15	.98–1.36
Emotional Labour	.08	1.08	.97–1.20
Social Anxiety	.37	1.45***	1.18–1.78
Partner Race * Emotional Labour	.14	1.15*	1.03–1.27

*Note*: Partner Race is coded as −1 = Same‐race interaction, 1 = Cross‐race interaction. Choosing to be anonymous or identifiable is coded as 1 = Anonymous, 0 = Identifiable. *p < .05. **p < .01. ***p < .001.

Abbreviation: CI, confidence interval.

**TABLE 9:** Simple slopes of codeswitching, emotional labour and burnout predicting anonymity by interaction partner.

	Codeswitching, emotional labour or burnout as moderator		Interaction partner as moderator	
	+1SD	−1SD	Same‐race	Cross‐race
Burnout	.97***	−.37	−.67*	.62*
Codeswitching	.83**	−.27	−.21	.87**
Emotional Labour	.73*	.16	−.20	.69*

*Note*: Entries represent log‐odds coefficients with positive values indicating a higher likelihood of choosing anonymity. *p < .05. **p < .01. ***p < .001.

## GENERAL DISCUSSION

In the present paper we consider why people might seek anonymity in an online interracial interaction. We make the case that Black people in majority‐White societies face substantial pressures during interracial interactions and therefore may be motivated to seek anonymity in an online interracial interaction, potentially to reduce or avoid these pressures. We do not find evidence for an overall effect whereby people seek anonymity in interracial interactions. Instead, we find that codeswitching, emotional labour and burnout are associated with seeking anonymity in online interracial interactions for Black people living in majority‐White societies. Burnout emerged as the most consistent predictor, while similar, although less consistent, associations were observed for codeswitching and emotional labour. Our mega‐analytic approach provided evidence for the reliability of these associations.

From a theoretical perspective, these findings can be situated within communication accommodation theory and SIDE. Communication accommodation theory highlights that communicative behaviours such as codeswitching and emotional labour are often strategic responses to an interaction, particularly for racially minoritized group members who face greater evaluative risk during interaction (Giles, 2008). Further, as SIDE suggests, anonymity does not eliminate group processes, but rather alters the identity frame, visibility and accountability that structure an interaction (Spears & Lea, 1994). Together these perspectives suggest that anonymity may be sought as a way of reorganizing the social conditions under which an interracial interaction occurs. Consistent with this view, our findings indicate that those who report greater performative burden during interracial interactions, particularly burnout, are more motivated to seek anonymity when anticipating online interracial contact.

We also compared whether Black and White people differed in the extent to which they experience codeswitching, emotional labour and burnout, and, in turn, seek anonymity. As expected, Black participants reported engaging in more codeswitching and emotional labour, felt greater burnout and were more likely to seek anonymity. This pattern of results is consistent with past work showing that racial minority and majority group members do not experience interracial contact in the same way (Bergsieker et al., 2010; Shelton et al., 2005). In Study 3, we find that those who were more aware of stigma associated with their race or experienced more day‐to‐day discrimination reported greater burnout during interactions with White people, and in part through this were more likely to want to be anonymous.

## IMPLICATIONS

The contact literature has traditionally assumed a model of contact characterized by face‐to‐face interactions. However, not all contact is experienced in this way. Indeed, there is a growing body of contact research that has examined how intergroup contact plays out online (e.g. Amichai‐Hamburger & McKenna, 2006; Imperato et al., 2021; White et al., 2020). The present research contributes to this work by engaging with the unique affordances that differentiate online from face‐to‐face environments (in this case the ability to be anonymous) that can impact the way in which people choose to engage in interracial interactions.

Anonymity can be used as a tool to alter how people want to be perceived in an interaction. People can choose what identifying information (both personal and social) they want to reveal or conceal (Bargh et al., 2002). In face‐to‐face interactions people rarely have the choice to interact under the veil of anonymity; in person, people (and their racial identities) are readily identifiable. However, it is easy to make a fake Instagram or Tumblr, remain muted on a voice chat while playing League of Legends, keep the camera turned off on Discord, or create a false identity on virtually any online platform. We are the first to show that this decision is not just a personal one; rather it is associated with group membership and intergroup experiences and attitudes. Our findings highlight the importance of understanding the unique affordances of the environment in which contact transpires, as well as when, how and why people use such affordances.

Our findings also highlight how responses associated with the performative pressures of interracial contact, such as burnout, are associated with the discrimination that minoritized group members experience in their day‐to‐day lives. Although these experiences may occur offline or online, tour work suggests that they may carry forward, potentially shaping how people choose to engage in subsequent online interactions.

More broadly, our results highlight that the burden borne in interracial interactions is not equal, at least when it comes to codeswitching, emotional labour and burnout. Our results paint a picture of interracial interactions where Black people in majority‐White contexts bear the brunt of the stress involved during interracial interactions. These results are consistent with past work highlighting the multifaceted effects of systemic and interpersonal racism on the interracial interactions of Black Americans (Evans & Moore, 2015; Grandey et al., 2019; McCluney et al., 2021; Sue et al., 2007). Much work on intergroup contact (including that which is online) primarily focuses on how contact might reduce prejudice (Pettigrew & Tropp, 2006; although see O'Donnell et al., 2025). What is less discussed, however, is that the onus is usually placed on racial minority group members to respond in certain ways to ensure that prejudice is reduced. Our findings raise the possibility that this responsibility might come at a psychological cost, potentially leading to the avoidance of future interactions.

## LIMITATIONS AND FUTURE DIRECTIONS

The present research examined one plausible pathway through which anonymity may become attractive in interracial interactions. However, because our focal analyses are correlational, we cannot draw causal conclusions about the direction of the associations between performative responses to interracial contact and the decision to seek anonymity. Although our findings are consistent with the interpretation that greater engagement in codeswitching, emotional labour and especially burnout motivates anonymity seeking, the reverse or reciprocal pattern is also theoretically plausible. Prior work indicates that anonymity is often adopted strategically to regulate behaviour and manage social risk within interactions (Nitschinsk, Tobin, & Vanman, 2025). From this perspective, seeking anonymity may not merely reflect prior experiences of performative burden, but may also shape subsequent interactional strategies, for example by reducing the perceived need to accommodate, self‐monitor or regulate emotional expression once personal identifiability is lowered.

This bidirectionality is consistent with both communication accommodation theory and SIDE. Communication accommodation theory suggests that accommodative behaviours are sensitive to audience expectations and evaluative risk, whereas SIDE emphasizes that anonymity can reorganize visibility, accountability and power within an interaction. Together, these frameworks imply that anonymity may function not only as an outcome of prior intergroup experiences, but also as an antecedent condition that alters how interracial interactions are navigated. For example, individuals who anticipate interacting anonymously may feel less compelled to codeswitch or engage in emotional labour, or may experience reduced burnout over time, even if group norms remain salient. Disentangling these possibilities requires designs that move beyond cross‐sectional self‐report.

Future research should therefore adopt experimental and longitudinal approaches to more precisely map these pathways. Experimental studies could manipulate identifiability in online interracial interactions to test whether anonymity causally reduces accommodative behaviour, emotional regulation or experienced strain, and whether these effects differ as a function of prior burnout or stigma‐related experiences. Longitudinal designs could examine whether repeated exposure to interracial interactions under conditions of anonymity predicts changes in burnout, avoidance or willingness to engage over time. In addition, qualitative or mixed‐method approaches could directly probe participants' motivations for seeking anonymity, including whether anonymity is perceived as a means of reducing performative pressure, managing identity salience or avoiding anticipated negative evaluation. Such work would also help distinguish anonymity seeking from outright avoidance, clarifying whether anonymity serves as a self‐protective strategy that enables engagement, or whether it represents an intermediate step towards disengagement from interracial interaction altogether.

Another limitation of this research is the use of hypothetical scenarios. In our studies participants were exposed to vignettes. The advantage of using vignettes is that they allowed us to manipulate the race of the interaction partner while holding all other features constant, providing a clean test of our hypotheses. Vignette designs are commonly used in intergroup contact research (e.g. Hayward et al., 2017; Reimer et al., 2021). However, they do not reflect how people respond in real‐life situations, which involve more complex social pressures, a myriad of personalizing information and varying degrees of anonymity. Furthermore, using a vignette design meant that participants were only responding to the vignette and were not actually avoiding or altering an intergroup interaction. To address these problems future research could explore when and why members of racially minoritized groups choose to be anonymous when playing online games with strangers, or keep their race hidden when participating in an online discussion forum or using social media.

Additionally, while we controlled for social anxiety in all models, we did not include a measure of intergroup anxiety. We chose to assess social anxiety because it captures a general tendency to experience discomfort in social interactions and has been shown to predict anonymity seeking online (Nitschinsk, Tobin, Varley, & Vanman, 2025). Future research, however, should examine whether intergroup anxiety explains additional variance in anonymity seeking beyond general social anxiety.

Finally, we focused solely on interactions between Black and White people living in majority‐White nations. Members of different racially minoritized groups have different relationships with one another, and indeed, with racial majority groups (Barlow et al., 2010). As such, it's unclear whether the relationship between codeswitching, emotional labour and burnout can be generalized across various intergroup dyads. Future research should investigate how engaging in codeswitching and emotional labour, and feeling burned out, relates to seeking anonymity differs across racial minority groups and across interaction partners.

## CONCLUSION

Interracial interactions are a vital part of life in any multicultural society but also carry significant pressures, especially for members of racially minoritized groups in majority‐White nations who face systemic and interpersonal discrimination. In online environments, such pressures may encourage the use of anonymity to control when race becomes a feature of an interaction. We found that Black people who reported engaging in more codeswitching and emotional labour while interacting with White people, and who felt greater burnout after interactions with White people, were more likely to seek anonymity in online interracial interactions. Further, Black participants reported engaging in more codeswitching and emotional labour and felt more burned out by interracial interactions than did White people. These responses to the performative demands of contact helped to explain a general pattern whereby Black participants wanted to be anonymous in online interracial interactions more so than did White people. Finally, both Black participants who reported experiencing more everyday discrimination and stigma consciousness were more likely to report feeling burned out by interracial interactions and to report engaging in more emotional labour during them. It is our hope that this research prompts further examinations of how, whether and when we engage in contact in digital spaces.

## AUTHOR CONTRIBUTIONS

**Lewis Nitschinsk:** Conceptualization; methodology; investigation; formal analysis; project administration; writing – review and editing; visualization; writing – original draft; data curation. **Melinda Hewett:** Conceptualization; writing – review and editing; investigation. **Audree Grand'Pierre:** Conceptualization; writing – review and editing; investigation. **Michael Thai:** Writing – review and editing; supervision; conceptualization. **Fiona Kate Barlow:** Conceptualization; methodology; data curation; supervision; writing – review and editing; investigation; formal analysis; resources.

## FUNDING INFORMATION

This work was supported by the Australian Research Council Discovery Grant scheme: DP220102606.

## CONFLICT OF INTEREST STATEMENT

None declared.

## ETHICS STATEMENT

All studies received ethics approval and were pre‐registered by the University of Queensland's Ethic Review Board. All participants gave their written informed consent before participating in the study.

## Supporting information


Data S1.

## Data Availability

All data and analysis code have been made publicly available using the Open Science Framework. The link can be accessed here: https://osf.io/fx95b/?view_only=2a8028999e5a48db8d1fcf65de50a904.

## Footnotes

^1^
An exploratory factor analysis indicated that all items on this scale load onto a single factor. See supplemental materials.

^2^
Participants also responded to two continuous items on a 7‐point scale: *If you were in this scenario, how likely is it that you would decide to be anonymous/identifiable?* (1 = *Very Unlikely*, 7 = *Very Likely*). We calculated a difference score for the likelihood of choosing anonymity versus identifiably. The pattern of results using both the dichotomous and difference score measure was consistent. For results, see supplemental materials.

^3^
Following Study 2 we ran an additional preregistered study trying to manipulate partner race via a photo. In this study there were no significant associations between any measured variable and seeking anonymity with people in the photos. We have moved this study to the supplemental materials. The supplemental materials include the original pre‐registration, as well as the rationale, methods and results for the study. Additionally, we provide a detailed discussion about why this study might not have worked.
