# Supplementary material for: Why might members of racially minoritized groups seek anonymity when interacting with White people online? Codeswitching, emotional labour and burnout
Source: Br J Soc Psychol. 2026 Mar 8;65(2):e70060. doi: 10.1111/bjso.70060 (PMC12967767; doi:10.1111/bjso.70060)
Supplement: Supplementary file 1 — Data S1. [file BJSO-65-0-s001.docx]

**Supplemental Materials**

**Links to Preregistrations and OSF:**

**OSF**

<https://osf.io/fx95b/?view_only=2a8028999e5a48db8d1fcf65de50a904>

**Preregistration Study 1**

<https://osf.io/dxb7y/?view_only=2f0eb62b360449bfae3fa8e8d3dcabeb>

**Preregistration Study 2**

<https://osf.io/2cfqb/?view_only=a5f0a4ddd5174eac93fcecf5ef49165a>

**Preregistration Study 3 and Study A**

<https://osf.io/my29n/?view_only=14062b800dc8405b80bdd05afe38a0f5>

**Study 1**

**List of All Variables**

1. Demographics
   1. Age
   2. Gender
   3. Education
   4. Relationship Status
   5. Interracial Relationship Status
2. % Time Spent in White Environments
3. Burnout (Maslach et al., 1986)
4. Surface Acting (Brotheridge & Lee, 2003)
5. Codeswitching (Durr & Wingfield, 2011; Evans & Moore, 2015; McCluney et al., 2021)
6. Intergroup Avoidance: In general, I would prefer to avoid White people?
7. The Online Anonymity Questionnaire (Nitschinsk et al., 2023)
8. Trait Self-Concept Clarity (Campbell et al., 1996)
9. Big Five Inventory Extra Small (Soto & John, 2017)
10. Self-Esteem (Rosenberg, 1965)
11. Social Anxiety (Scheier & Carver, 1985)
12. Vignette

**Exploratory Factor Analysis for Codeswitching**

**Table A**

*Factor loadings of the exploratory factor analysis*

| Item | *Factor* |
| --- | --- |
| I try to paint my racial group in a positive light. | 0.583 |
| I go out of my way to make sure the interaction is a positive one. | 0.769 |
| I feel overly conscious of my own behavior. | 0.687 |
| I actively monitor my own behavior. | 0.775 |
| I avoid race-sensitive topics. | 0.426 |
| I am very careful with how I act. | 0.833 |
| I try to smile more. | 0.770 |
| I alter the way I speak. | 0.472 |
| I try to voice my opinion in the calmest way possible. | 0.639 |

*Note.* Factor loadings reflect values from the pattern matrix.

**Preregistered Analyses Not Included in the Main Manuscript**

**Table B**

*Multiple regression for interaction partner ethnicity, social anxiety, and either burnout, codeswitching, or emotional labour as predictors of choosing to be anonymous during a social interaction (continuous dependent variable).*

| Predictors | β | *SE* | 95% *CI* |
| --- | --- | --- | --- |
| Panel A: Codeswitching |  |  |  |
| Intercept | -0.06 | 0.08 | -0.22 – 0.10 |
| Partner Race | 0.12 | 0.11 | -0.10 – 0.34 |
| Codeswitching | -0.05 | 0.09 | -0.22 – 0.12 |
| Emotional Labour | -0.00 | 0.08 | -0.16 – 0.15 |
| Burnout | 0.02 | 0.07 | -0.11 – 0.15 |
| Social Anxiety | 0.22 ^***^ | 0.06 | 0.11 – 0.34 |
| Partner Race* Codeswitching | 0.25 ^*^ | 0.11 | 0.03 – 0.46 |
| Panel B: Emotional Labour |  |  |  |
| Intercept | -0.06 | 0.08 | -0.22 – 0.09 |
| Partner Race | 0.12 | 0.11 | -0.10 – 0.34 |
| Codeswitching | 0.07 | 0.07 | -0.06 – 0.20 |
| Emotional Labour | -0.03 | 0.09 | -0.20 – 0.15 |
| Burnout | 0.03 | 0.07 | -0.10 – 0.16 |
| Social Anxiety | 0.22 ^***^ | 0.06 | 0.10 – 0.33 |
| Partner Race * Emotional Labour | 0.01 | 0.11 | -0.21 – 0.23 |
| Panel C: Burnout |  |  |  |
| Intercept | -0.05 | 0.08 | -0.21 – 0.10 |
| Partner Race | 0.12 | 0.11 | -0.09 – 0.34 |
| Codeswitching | 0.07 | 0.07 | -0.06 – 0.20 |
| Emotional Labour | -0.01 | 0.08 | -0.17 – 0.14 |
| Burnout | -0.09 | 0.08 | -0.25 – 0.08 |
| Social Anxiety | 0.21 ^***^ | 0.06 | 0.09 – 0.32 |
| Partner Race* Burnout | 0.26 ^*^ | 0.11 | 0.04 – 0.47 |

*Note.* Interaction partner ethnicity is coded as -1 = Black interaction partner, 1 = White interaction partner. SE = Standard Error. **p* < .05. ***p* < .01. ****p* < .001.

**Table C**

*Logistic regression for partner race, social anxiety, codeswitching, emotional labour, and burnout as predictors of choosing to be anonymous or identifiable during a social interaction.*

| Predictors | *Wald* | *Odds Ratio* | 95% *CI* |
| --- | --- | --- | --- |
| Intercept | -.90 | 0.41 ^***^ | 0.25 – 0.66 |
| Partner Race | .08 | 1.08 | 0.86 – 1.37 |
| Burnout | .01 | 0.11 | -0.10 – 0.34 |
| Codeswitching | .15 | 1.01 | 0.75 – 1.36 |
| Emotional Labour | -.03 | 0.97 | 0.80 – 1.19 |
| Social Anxiety | .38 | 1.47 ^*^ | 1.09 – 1.97 |
| Partner Race * Burnout | .39 | 1.47 ^*^ | 1.10 – 1.99 |
| Partner Race* Codeswitching | .28 | 1.32 ^*^ | 1.06 – 1.65 |
| Partner Race * Emotional Labour | -.15 | 0.86 | 0.71 – 1.04 |

*Note.* Interaction partner ethnicity is coded as -1 = Black interaction partner, 1 = White interaction partner. SE = Standard Error. **p* < .05. ***p* < .01. ****p* < .001.

**Study 2**

**Explanation for Preregistered Analyses not Included in the Main Manuscript**

We did not include our pre-registered three-way interaction between participant race, partner race, and either burnout, codeswitching, or emotional labour due to limited statistical power. Our total sample of 560 participants is too small to robustly test a three-way interaction that may follow an attenuation or knockout pattern. Such a pattern would suggest that a predictor (e.g., codeswitching) affects anonymity only for one group (e.g., Black participants interacting with a White partner), and not at all for others, essentially “knocking out” the effect in other subgroups. Detecting such nuanced patterns requires very large sample sizes (potentially up to 14 times larger than ours) to achieve adequate power (Baranger, 2023; Da Silva Frost & Ledgerwood, 2020).

We additionally preregistered to investigate how motivations to respond without prejudice and warmth and competence motivations were associated with seeking anonymity. Whereas the results were largely in line with our predictions, due to space constraints we have moved these results to the supplementary materials.

**Table D**

*Logistic regression for race, interaction partner ethnicity, social anxiety, and either burnout, codeswitching, or emotional labour as predictors of choosing to be anonymous during a social interaction.*

| Predictors | Wald | *Odds Ratio* | *95% CI* |
| --- | --- | --- | --- |
| Panel A: Codeswitching |  |  |  |
| Intercept | -0.25** | 0.78 | 0.65 – 0.93 |
| Interaction Partner Ethnicity | -0.07 | 0.93 | 0.78 – 1.12 |
| Race | -0.27** | 0.76 | 0.63 – 0.92 |
| Codeswitching | 0.15* | 1.16 | 1.00 – 1.34 |
| Social Anxiety | 0.41*** | 1.50 | 1.22 – 1.85 |
| Interaction Partner Ethnicity * Race | -0.21* | 0.81 | 0.68 – 0.97 |
| Interaction Partner Ethnicity * Codeswitching | 0.12 | 1.12 | 0.97 – 1.30 |
| Race * Codeswitching | -0.03 | 1.03 | 0.89 – 1.19 |
| Interaction Partner Ethnicity * Codeswitching * Race | -0.06 | 0.94 | 0.81 – 1.09 |
| Panel B: Emotional Labour |  |  |  |
| Intercept | -0.22** | 0.80 | 0.67 – 0.96 |
| Interaction Partner Ethnicity | -0.07 | 0.94 | 0.78 – 1.12 |
| Race | -0.25* | 0.78 | 0.64 – 0.94 |
| Emotional Labour | 0.21** | 1.23 | 1.08 – 1.40 |
| Social Anxiety | 0.39*** | 1.47 | 1.19 – 1.83 |
| Interaction Partner Ethnicity * Race | -0.18* | 0.83 | 0.69 – 1.00 |
| Interaction Partner Ethnicity * Emotional Labour | 0.18** | 1.20 | 1.05 – 1.37 |
| Race * Emotional Labour | 0.08 | 1.08 | 0.95 – 1.24 |
| Interaction Partner Ethnicity * Emotional Labour * Race | -0.04 | 0.96 | 0.84 – 1.10 |
| Panel C: Burnout |  |  |  |
| Intercept | -0.15 | 0.86 | 0.71 – 1.05 |
| Interaction Partner Ethnicity | -0.09 | 0.92 | 0.75 – 1.11 |
| Race | -0.25* | 0.78 | 0.64 – 0.96 |
| Burnout | 0.26 | 1.30 | 1.00 – 1.72 |
| Social Anxiety | 0.41*** | 1.50 | 1.21 – 1.87 |
| Interaction Partner Ethnicity * Race | -0.19 | 0.83 | 0.68 – 1.01 |
| Interaction Partner Ethnicity * Burnout | 0.23 | 1.26 | 0.96 – 1.64 |
| Race * Burnout | 0.32* | 1.38 | 1.07 – 1.83 |
| Interaction Partner Ethnicity * Burnout * Race | -0.13 | 0.88 | 0.67 – 1.14 |

*Note.* Interaction partner ethnicity is coded as -1 = Black interaction partner, 1 = White interaction partner. **p* < .05. ***p* < .01. ****p* < .001.

**Table E**

*Descriptive statistics, scale reliabilities, and bivariate correlations between internal and external motivations to respond without prejudice, warmth motivations, competence motivations, and focal predictors in Study 2*

|  | *M (SD)* | α | 1 | 2 | 3 | 4 | 5 | 6 | 7 | 8 | 9 |
| --- | --- | --- | --- | --- | --- | --- | --- | --- | --- | --- | --- |
| 1. Race |  |  |  |  |  |  |  |  |  |  |  |
| 1. Anonymity | 0.56 (0.49) |  | -.119^**^ |  |  |  |  |  |  |  |  |
| 1. Burnout | 1.61 (0.84) | .97 | -.304^***^ | .115^**^ |  |  |  |  |  |  |  |
| 1. Codeswitching | 4.10 (1.29) | .89 | -.303^***^ | .167^***^ | .604^***^ |  |  |  |  |  |  |
| 1. Emotional Labour | 2.79 (1.45) | .87 | -.395^***^ | .124^**^ | .336^***^ | .553^***^ |  |  |  |  |  |
| 1. Social Anxiety | 1.62 (0.87) | .89 | .250^***^ | .123^**^ | -.062 | .046 | .113^**^ |  |  |  |  |
| 1. Internal Motivations | 5.37 (1.30) | .86 | .288^***^ | -.119^**^ | -.012 | -.330^***^ | -.429^***^ | -.018 |  |  |  |
| 1. External Motivations | 3.36 (1.34) | .82 | .087^*^ | .155^***^ | .432^***^ | .330^***^ | .125^**^ | .103^*^ | .059 |  |  |
| 1. Warmth Motivations | 5.43 (1.25) | .93 | .099^*^ | -.054 | .339^***^ | -.056 | -.155^***^ | -.056 | .414^***^ | .230^***^ |  |
| 1. Competence Motivations | 5.40 (1.29) | .93 | -.155^***^ | -.016 | .401^***^ | .096^*^ | .027 | -.110^**^ | .238^***^ | .208^***^ | .794^***^ |

*Note. N* = 560, α = Cronbach’s alpha. Point biserial correlations were conducted between predictor variables and race or choosing to be anonymous. **p* < .05. ***p* < .01. ****p* < .001.

**Moderated Regression Analyses Results**

Due to space constraints, we included moderated regression analyses and simple slope analyses in the supplemental materials. Here we show regressions where burnout, codeswitching, and emotional labour and their respective interactions with partner race are included in separate models (Table F), as well as models where all covariates and their respective interactions are included in the same model (Table G).

**Table F**

*Logistic regressions for partner race, social anxiety, codeswitching, emotional labour, and burnout as predictors of choosing to be anonymous or identifiable during a social interaction.*

|  | White Participants | | | Black Participants | | |
| --- | --- | --- | --- | --- | --- | --- |
| Predictors | *Wald* | *Odds Ratio* | 95% *CI* | *Wald* | *Odds Ratio* | 95% *CI* |
| Panel A: Codeswitching |  |  |  |  |  |  |
| Intercept | -0.50 | 0.60 ^***^ | 0.46 – 0.79 | 0.04 | 1.04 | 0.80 – 1.36 |
| Partner Race | -0.28 | 0.76 ^*^ | 0.59 – 0.98 | 0.14 | 1.15 | 0.89 – 1.49 |
| Codeswitching | 0.17 | 1.19 | 0.96 – 1.48 | 0.12 | 1.12 | 0.93 – 1.36 |
| Social Anxiety | 0.34 | 1.40 ^*^ | 1.04 – 1.91 | 0.47 | 1.59 ^**^ | 1.20 – 2.13 |
| Partner Race* Codeswitching | 0.06 | 1.06 | 0.85 – 1.31 | 0.18 | 1.19 | 0.99 – 1.45 |
| Panel B: Emotional Labour |  |  |  |  |  |  |
| Intercept | -0.46 | 0.63 ^***^ | 0.48 – 0.82 | 0.03 | 1.03 | 0.79 – 1.36 |
| Partner Race | -0.25 | 0.78 | 0.60 – 1.01 | 0.12 | 1.13 | 0.87 – 1.46 |
| Emotional Labour | 0.28 | 1.32 ^**^ | 1.08 – 1.64 | 0.12 | 1.13 | 0.96 – 1.34 |
| Social Anxiety | 0.35 | 1.42 ^*^ | 1.05 – 1.95 | 0.42 | 1.52 ^**^ | 1.13 – 2.05 |
| Partner Race * Emotional Labour | 0.15 | 1.16 | 0.94 – 1.43 | 0.22 | 1.25 ^**^ | 1.06 – 1.47 |
| Panel C: Burnout |  |  |  |  |  |  |
| Intercept | -0.37 | 0.69 ^*^ | 0.51 – 0.94 | 0.13 | 1.14 | 0.86 – 1.51 |
| Partner Race | -0.27 | 0.76 | 0.57 – 1.01 | 0.11 | 1.11 | 0.86 – 1.45 |
| Burnout | 0.59 | 1.81 ^**^ | 1.18 – 2.93 | -0.09 | 0.91 | 0.68 – 1.22 |
| Social Anxiety | 0.31 | 1.36 | 1.00 – 1.86 | 0.50 | 1.65 ^**^ | 1.22 – 2.26 |
| Partner Race * Burnout | 0.10 | 1.10 | 0.69 – 1.73 | 0.36 | 1.44 ^*^ | 1.10 – 1.91 |

*Note.* CI = Confidence Interval. Partner Race is coded as -1 = same-race partner, 1 = different-race partner. Choosing to be anonymous or identifiable is coded as 1 = Anonymous, 0 = Identifiable. **p* < .05. ***p* < .01. ****p* < .001.

**Simple Slopes Analysis For Significant Interactions in Table F**

For models that included only Black participants, we found a significant interaction between partner race and emotional labour (see Figure 4). Participants who reported engaging in more emotional labour while interacting with White people (+1SD) were more likely to seek anonymity in cross-race interactions compared to same-race interactions (*b* = 1.13, *p* = .004), but no difference was found for people who engaged in less emotional labour (-1SD; *b* = -.26, *p* = .720). When examining interaction partner race as the moderator follow up tests showed that the more people engaged in emotional labour in cross-race interactions the more they wanted online anonymity when interacting with Black (*b* = 1.07, *p* = .012), but not White people (*b* = -.31, *p* = .622).

We also found a significant interaction between burnout and partner race. Participants who reported feeling more burnout while interacting with White people (+1SD) were more likely to seek anonymity in cross-race interactions compared to same-race interactions (*b* = 1.13, *p* = .004), but no difference was found for people who felt less burnout (-1SD; *b* = -.20, *p* = .815). Examining the simple slopes the other way, people higher in burnout were not more likely to seek anonymity in a cross-race interaction (*b* = .50, *p* = .362) but were more likely to seek identifiability in a same-race interaction (*b* = -.83, *p* = .045). There was no interaction between partner race and codeswitching.

**Table G**

*Logistic regression for partner race, social anxiety, codeswitching, emotional labour, and burnout as predictors of choosing to be anonymous or identifiable during a social interaction for White and Black participants.*

| Predictors | *Wald* | *Odds Ratio* | 95% *CI* |
| --- | --- | --- | --- |
| Panel A: White Participants |  |  |  |
| Intercept | -.37 | 0.69 ^*^ | 0.51 – 0.94 |
| Partner Race | -.28 | 0.75 | 0.56 – 1.01 |
| Burnout | .38 | 1.46 | 0.91 – 2.46 |
| Codeswitching | -.01 | 0.99 | 0.75 – 1.30 |
| Emotional Labour | .21 | 1.24 | 0.93 – 1.65 |
| Social Anxiety | .33 | 1.38 ^*^ | 1.02 – 1.90 |
| Partner Race * Burnout | -.07 | 0.93 | 0.56 – 1.52 |
| Partner Race* Codeswitching | -.04 | 0.96 | 0.73 – 1.26 |
| Partner Race * Emotional Labour | .16 | 1.18 | 0.89 – 1.57 |
| Panel B: Black Participants |  |  |  |
| Intercept | .08 | 1.09 | 0.81 – 1.45 |
| Partner Race | .06 | 1.07 | 0.81 – 1.40 |
| Burnout | -.26 | 0.77 | 0.55 – 1.08 |
| Codeswitching | .05 | 1.05 | 0.83 – 1.33 |
| Emotional Labour | .17 | 1.19 | 0.95 – 1.49 |
| Social Anxiety | .49 | 1.63 ^**^ | 1.20 – 2.25 |
| Partner Race * Burnout | .24 | 1.27 | 0.92 – 1.76 |
| Partner Race* Codeswitching | .07 | 1.07 | 0.85 – 1.35 |
| Partner Race * Emotional Labour | .12 | 1.13 | 0.91 – 1.41 |

*Note.* Interaction partner ethnicity is coded as -1 = Black interaction partner, 1 = White interaction partner. SE = Standard Error. **p* < .05. ***p* < .01. ****p* < .001.

**Parallel Mediation Indirect Effects**

**Table H**

*Conditional indirect effects for the relationship between race and seeking anonymity or identifiability when interacting with someone from the same racial group or a different racial group via burnout, codeswitching, and emotional labour.*

| Mediator | Condition | *Effect* | *SE* | 95% *CI* |
| --- | --- | --- | --- | --- |
| Burnout | Same-Race | -.06 | .07 | -.22 – .07 |
|  | Cross-Race | .03 | .08 | -.13 – .20 |
| Codeswitching | Same-Race | .01 | .05 | -.09 – .11 |
|  | Cross-Race | .02 | .05 | -.09 – .12 |
| Emotional Labour | Same-Race | .01 | .06 | -.10 – .13 |
|  | Cross-Race | .16* | .07 | .04 – .32 |

*Note.* * Significant indirect effect.

**Study 3**

**Parallel Mediation Indirect Effects**

**Table I**

*Indirect effects for parallel mediation between stigma consciousness and codeswitching, emotional labour, and burnout, on seeking anonymity when interacting with a White person.*

| Predictors | β | SE | *p* | 95% CI Upper | 95% CI Lower |
| --- | --- | --- | --- | --- | --- |
| Indirect Effect Burnout | .04 | .04 | .214 | -.03 | .11 |
| Indirect Effect Codeswitching | -.01 | .01 | .516 | -.03 | .02 |
| Indirect Effect Emotional Labour | .03 | .02 | .094 | -.01 | .06 |
| Indirect Effect Total | .07 | .04 | .083 | -.01 | .14 |

*Note.* SE = Standard Error. CI = Confidence Interval.

**Table J**

*Parallel mediation between emotional labour and codeswitching, emotional labour, and burnout, on seeking anonymity when interacting with a White person.*

| Predictors | β | SE | *p* | 95% CI Upper | 95% CI Lower |
| --- | --- | --- | --- | --- | --- |
| Indirect Effect Burnout | -.01 | .06 | .844 | -.14 | .11 |
| Indirect Effect Codeswitching | .25 | .07 | < .001 | .12 | .39 |
| Indirect Effect Emotional Labour | .54 | .05 | < .001 | .45 | .63 |
| Indirect Effect Total | .07 | .04 | .089 | -.01 | .15 |

*Note.* SE = Standard Error. CI = Confidence Interval.

**Mega Analysis**

**Table K**

*Logistic regression for partner race, social anxiety, codeswitching, emotional labour, and burnout as predictors of choosing to be anonymous or identifiable during a social interaction for White and Black participants.*

| Predictors | *Wald* | *Odds Ratio* | 95% *CI* |
| --- | --- | --- | --- |
| Panel A: White Participants |  |  |  |
| Intercept | -.20 | 0.82 | 0.63 – 1.06 |
| Partner Race | .14 | 1.15 | 0.97 – 1.37 |
| Burnout | -.08 | 0.92 | 0.73 – 1.14 |
| Codeswitching | .11 | 1.11 | 0.95 – 1.30 |
| Emotional Labour | .06 | 1.06 | 0.92 – 1.22 |
| Social Anxiety | .41 | 1.50 ^***^ | 1.22 – 1.85 |
| Partner Race * Burnout | .33 | 1.40 ^**^ | 1.12 – 1.73 |
| Partner Race* Codeswitching | .18 | 1.20 ^*^ | 1.03 – 1.41 |
| Partner Race * Emotional Labour | -.04 | 0.97 | 0.84 – 1.11 |

*Note.* Interaction partner ethnicity is coded as -1 = Black interaction partner, 1 = White interaction partner. SE = Standard Error. **p* < .05. ***p* < .01. ****p* < .001.

**Study A**

In Study A, we again test the theoretical model put forward in Study 3. However, in this study we have one further change. Instead of manipulating the race of participants’ interaction partner directly (i.e., telling them to imagine a Black or White person). In Study A, we aim to manipulate interaction partner race more subtly, via a photo. For this study we preregistered the design, planned sample size, inclusion/exclusion criteria, and planned analyses.

**Method**

***Participants and Design***

A power analysis for a linear multiple regression (fixed model, *R*^2^ deviation from zero, alpha = .05, predictors = 3) revealed that 195 participants would provide 95% power to detect small to medium effect sizes (*f*^2^ = .09). We recruited 205 participants. Nineteen participants were excluded for failing to pass either an attention check or a manipulation check (final *N* = 186, 109 men, 175 women, 1 nonbinary, 1 prefer not to say, *M*_age_ = 35.2, *SD* = 11.3, *Range* = 18-71). A sensitivity analysis using the same conditions as above indicated that with this sample size our minimum detectable effect size was *f*^2^ = .09. We recruited Prolific workers in the USA, the UK, Canada, Australia, and New Zealand. We screened participants so that only participants who said they were Black in response to the question “*What ethnic group do you belong to?”* could complete the study.

***Measures and Procedures***

Participants completed the same codeswitching, emotional labour, burnout, stigma-consciousness, and everyday discrimination scales that were used in Study 3.

Participants then completed a similar hypothetical scenario to that used in Studies 1 – 3. All interaction partners in this scenario were White, however, and participants were shown an image of a White male with a neutral expression, instead of being explicitly told the race of the person they were to interact with. Images of White males were taken from the Chicago Face Database (Ma et al., 2015). Participants were randomly shown one of 50 potential images. We chose 50 images as this allowed for variance in age, physical measurements, and subjective measures.

**Data Analysis**

Bivariate correlations were conducted as in Studies 1 – 3. Additionally, we preregistered a series of mediations. Stigma consciousness and everyday discrimination were included as predictor variables. Either codeswitching, emotional labour, or burnout were included as mediators. Our dependent variable was choosing to seek anonymity or identifiability. Social anxiety was included as a covariate in all models.

**Results**

For descriptive statistics, scale reliabilities, and correlations, see Table 5. Fifty-four percent of participants chose to be anonymous. On a bivariate level, stigma consciousness was associated with seeking anonymity when shown an image of a White male interaction partner. No other variables were significantly associated with seeking anonymity. Stigma consciousness and everyday discrimination were positively associated with burnout, codeswitching, and emotional labour. Due to the nonsignificant correlations between burnout, codeswitching, emotional labour and seeking anonymity, preregistered mediations were by default nonsignificant.

**Table L**

*Descriptive statistics, scale reliabilities, and correlations between focal predictors in Study A*

|  | *M (SD)* | α | 1 | 2 | 3 | 4 | 5 | 6 |
| --- | --- | --- | --- | --- | --- | --- | --- | --- |
| 1. Anonymity | 0.45 (0.49) |  |  |  |  |  |  |  |
| 1. Burnout | 2.14 (0.99) | 0.97 | .13 |  |  |  |  |  |
| 1. Codeswitching | 3.93 (1.20) | 0.88 | .02 | .27^***^ |  |  |  |  |
| 1. Emotional Labour | 3.66 (1.54) | 0.82 | .07 | .48^***^ | .53^***^ |  |  |  |
| 1. Social Anxiety | 1.54 (0.84) | 0.86 | .13 | .22^**^ | .01 | .30^***^ |  |  |
| 1. Stigma Consciousness | 4.72 (1.09) | 0.86 | .18^*^ | .56^***^ | .25^***^ | .38^***^ | .30^***^ |  |
| 1. Everyday Discrimination | 2.58 (1.14) | 0.94 | .11 | .60^***^ | .34^***^ | .37^***^ | .05 | .44^***^ |

*Note. N* = 186, α = Cronbach’s alpha. Point biserial correlations were conducted between predictor variables and choosing to be anonymous. Choosing to be anonymous or identifiable is coded as 1 = Anonymous, 0 = Identifiable. **p* < .05. ***p* < .01. ****p* < .001.

**Discussion**

As we predicted, stigma consciousness and experiences of everyday discrimination were each positive and significantly associated with engagement in codeswitching and emotional labour, as well as feeling burned out after interacting with White people. In contrast to Studies 1, 2 and 3, however, these variables were not associated with a desire to seek anonymity.

Three explanations for this discrepancy can be immediately discounted. First, it is not the case that the amount of people seeking anonymity dramatically shifted (e.g., 59% of people sought anonymity in Study 3, 50% sought anonymity in Study 2). Second, although we showed participants one of 50 potential faces, we found that only an additional 3% of variance was explained when we included the images as a random intercept. Third, we found no difference in the significance of bivariate correlations across male and female participants (See R-Markdown).

In retrospect, this discrepancy is perhaps not surprising. Studies 1-3 used a minimal group paradigm approach, which has been shown to be effective in understanding intergroup processes in a variety of contexts (Otten, 2016; Tajfel & Turner, 1979). When we changed our manipulation to a photo, participants were given more contextual information about their interaction partner, making the interaction one that is no longer simply intergroup (Tsao & Livingstone, 2008). People form impressions, evaluate, and make judgements about other people from a variety of physical and emotional qualities (Zebrowitz & Montepare, 2008). These qualities may influence people’s choice to seek anonymity, beyond the race of their interaction partner in the photo.

However, other explanations for our lack of effects are also possible. As the effects we found in Studies 1-3 were modest, a larger sample size may be needed to uncover these effects when race is displayed as a subtle visual cue. Alternatively, it is possible that the faces, which were all male, and all neutral, were interpreted in a way that led some people who generally feel burned out after interracial interactions, for example, to feel compelled to become identifiable. Such a pattern would eliminate the main effects observed in Studies 1-3. In general, men are seen as more racist than women (Kwate & Goodman, 2015; Liang et al., 2011) and are interpreted as more threatening (Archer & Coyne, 2005). Further, neutral facial expressions can be interpreted in a mix of ways (Pfaltz et al., 2019). It is possible that with a wider variety of differently gendered and expressed faces we would see a different pattern of results emerge.

In sum, how race intersects with other salient features such as age, gender, emotional expression, and attractiveness, when people from different groups interact remains understudied (Kubota & Ito, 2007). As such, these findings provide compelling avenues for future research to take a more holistic approach when understanding how different cues affect interracial approach vs avoidance, and in the online space, whether people choose to be identifiable or anonymous.

**Study A Demographic Statistics**

***Education***

Less than High School = 4, High School or Equivalent = 16, Some University = 31, University Degree = 91, Masters Degree = 40, Doctoral Degree = 3, Professional Degree = 1.

***Relationship Status***

Single = 79, Dating = 44, Married/In a De Facto Relationship = 52, Separated or Divorced = 7, Widowed = 2, NA = 2.

**References**

Baranger, D. A. A., Finsaas, M. C., Goldstein, B. L., Vize, C. E., Lynam, D. R., & Olino, T. M. (2023). Tutorial: Power Analyses for Interaction Effects in Cross-Sectional Regressions. *Advances in Methods and Practices in Psychological Science*, *6*(3), 25152459231187531. <https://doi.org/10.1177/25152459231187531>

da Silva Frost, A., & Ledgerwood, A. (2020). Calibrate your confidence in research findings: A tutorial on improving research methods and practices. *Journal of Pacific Rim Psychology*, *14*, e14. <https://doi.org/10.1017/prp.2020.7>

Ma, D. S., Correll, J., & Wittenbrink, B. (2015). The Chicago face database: A free stimulus set of faces and norming data. *Behavior Research Methods*, *47*(4), 1122–1135. https://doi.org/10.3758/s13428-014-0532-5
